# Primary Prevention of Cardiovascular Disease at Community Clinics in the State of Sao Paulo, Brazil: Results from the Epidemiological Information Study of Communities

**DOI:** 10.5334/gh.1203

**Published:** 2023-05-05

**Authors:** Henrique Andrade R. Fonseca, Maria Cristina O. Izar, Luciano F. Drager, Ibraim M. Pinto, José Francisco K. Saraiva, João Fernando Monteiro Ferreira, Álvaro Avezum, Francisco Antonio Fonseca, Otávio Berwanger

**Affiliations:** 1 Research Center, Cardiology Society of the State of São Paulo –SOCESP, São Paulo, Brazil; 2 Hospital Albert Einstein, São Paulo, Brazil; 3 Cardiology Division, Department of Medicine, Universidade Federal de São Paulo, São Paulo, Brazil; 4 Instituto do Coração (InCor), Hospital das Clínicas HCFMUSP, Faculdade de Medicina, Universidade de Sao Paulo, Brazil; 5 Centro de Cardiologia, Hospital Sírio Libanês, Brazil; 6 Instituto Dante Pazzanese de Cardiologia, São Paulo, Brazil; 7 Pontifícia Universidade Católica de Campinas, Campinas, Brazil; 8 International Research Center, Hospital Alemão Oswaldo Cruz, São Paulo, Brazil

**Keywords:** hypertension, diabetes mellitus, dyslipidemia, primary cardiovascular prevention, health communities

## Abstract

**Background:**

Primary prevention of cardiovascular disease (CVD) remains a major challenge, especially in communities of low- and middle-income countries with poor medical assistance influenced by distinct local, financial, infrastructural, and resource-related factors.

**Objective:**

This a community-based study aimed to determine the proportion and prevalence of uncontrolled cardiovascular risk factors (CRF) in Brazilian communities.

**Methods:**

The EPICO study was an observational, cross-sectional, and community clinic-based study. Subjects were living in Brazilian communities and were of both sexes and ≥18 years old, without a history of a stroke or myocardial infarction but presenting at least one of the following cardiovascular risk factors: hypertension, diabetes mellitus and hypercholesterolemia. The study was carried out in Brazil, including 322 basic health units (BHU) in 32 cities.

**Results:**

A total of 7,724 subjects with at least one CRF were evaluated, and one clinical visit was performed. Mean age was 59.2 years-old (53.7% were >60 years old). A total of 66.7% were women. Of the total, 96.2% had hypertension, 78.8% had diabetes mellitus type II, 71.1% had dyslipidemia, and 76.6% of patients were overweight/obese. Controlled hypertension (defined by <130/80 mmHg or <140/90 mmHg) was observed in 34.9% and 55.5% patients among respective criteria, the rates of controlled blood glucose in patients taking antidiabetic medications was 29.5%, and among those with documented dyslipidemia who received any lipid-lowering medication, only 13.9% had LDL-c on target. For patients presenting three CRF less than 1.9% had LDL-c < 100 mg/dL once their BP and blood glucose were on target. High education level as associated with blood pressure (BP) target of less than 130 / 80mm Hg. The glucose and LDL-c levels on target were associated with the presence of hypertension and diabetes mellitus.

**Conclusion:**

In Brazilian community clinics, regarding most patients in primary prevention, the CRF such as BP, blood glucose, and lipid levels are poorly controlled, with a majority of patients not achieving guidelines/recommendations.

## 1. Introduction

Primary prevention of cardiovascular disease (CVD) remains a major challenge, especially in communities with poor access to medical assistance influenced by distinct local, financial, infrastructural, and resource-related factors [1]. The major risk factors for atherosclerosis, including hypertension, diabetes mellitus, and dyslipidemia, remain emerging public health problems globally, particularly in low- and middle-income countries with many people living in communities [2].

Randomized, controlled trials have shown that pharmacological and non-pharmacological therapies focusing on arterial hypertension, dyslipidemia, or diabetes may prevent cardiovascular events. However, in real-world clinical practice, patients have multiple uncontrolled risk factors requiring combined interventions [3,4]. Therefore, more effort is necessary to improve the primary prevention of cardiovascular events. Efficient risk-factor control has recently been examined in large registry studies in different countries and has shown great potential to reduce cardiovascular events [5].

Brazil has many family health strategies, consisting of a physician, nurses, and community health workers, taking care of its communities. These basic health care units provide medical assistance for non-communicable and infectious diseases, especially hypertension and diabetes mellitus through the HiperDia program [6].

This community clinic-based study aimed to determine the proportion and prevalence of uncontrolled CVD risk factors in Brazilian community clinics.

## 2. Methods

### 2.1 Study design

The Epidemiological Information Study of Communities (EPICO) was an observational, cross-sectional, community clinic-based study to evaluate the management of cardiovascular risk factors (CRFs) for primary CVD prevention among subjects living in Brazilian communities. The study was carried out in Brazil, including 322 basic health units (BHUs) in 32 cities of the state of São Paulo.

### 2.2 Study population

The study included subjects of both sexes who were 18 years or older; had no history of a stroke or myocardial infarction but previously presented at least one of the following CRFs: hypertension, type 2 diabetes, or dyslipidemia (definitions are provided in the next section) as confirmed by BHU medical staff; and were included in any local program for CRF control.

The subjects were invited to community clinics and interviewed by the local medical staff as per protocol training. Laboratory data were collected from medical records from the last 6 months before the interview.

### 2.3 Study procedures and data collection

After inviting the patients in the community around the BHU for clinical and physical examinations, data were collected by a trained nurse and medical staff. Data on the medical diagnosis, treatments, laboratory results, and lifestyle of subjects with at least one CRF were collected via interviews.

Lifestyle habits (smoking, physical activity, and eating habits) were evaluated in the interviews. Smoking habits were defined as self-reported smoking, former smoking (at least 1 year without smoking), or never smoking. Physical inactivity was defined as less than 150 minutes per week of moderate-intensity activity or 75 minutes per week of high-intensity activity. The physical activity target was defined as moderate physical activity for at least 30 minutes, on average, five times a week. Fruit and vegetable consumptions were defined as daily consumption for a minimum of 6 days a week.

Anthropometric evaluations were performed through measurements of height and weight to define the body mass index (BMI). Overweight was defined as a BMI ≥ 25 and < 30 kg/m² and obesity as a BMI ≥ 30 kg/m². Waist circumference was measured using an inelastic tape placed horizontally in the mid-axillary line midway between the lowest rim of the rib cage and the tip of the hip bone, with the patient standing. Abdominal obesity was defined as a waist circumference of ≥ 88 cm for women and ≥ 102 cm for men [7].

Blood pressure (BP) was measured three times during the clinical visit of the study using an aneroid sphygmomanometer, with the arm’s cuff adjusted for size and shape. All measurements were performed by trained physicians at the BHU. Uncontrolled BP was defined by two criteria: systolic BP (SBP) ≥ 130 mmHg or ≥ 140 mmHg and/or diastolic BP (DBP) ≥ 80 mmHg or 90 mmHg, based on the guidelines of the American Heart Association [8] and the European Society of Cardiology/European Society of Hypertension [9].

Laboratory data were collected from medical records from the past 6 months. Low-density lipoprotein-cholesterol (LDL-c), high-density lipoprotein-cholesterol (HDL-c), total cholesterol, and triglycerides were used to evaluate dyslipidemia. The level of LDL-c was calculated using Friedewald’s formula when triglycerides were < 400 mg/dL [10]. Blood glucose analyses were performed using an automated enzymatic test. The certifications for Good Clinical Laboratory Practices of the laboratories used for biochemistry analyses in the study were noted. Data derived from uncertified laboratories were excluded.

### 2.4 Data management

All data were collected by BHU professionals trained in CRF studies, and the results were sent to a core unit at the Research Center of the Cardiology Society of the State of São Paulo (SOCESP) to be reviewed for completeness, consistency, and accuracy and for recording under the coordination of the SOCESP. The Research Center of the SOCESP provided training for data collection and revision before receiving the data.

### 2.5 Medication use

The interviews collected information on the use of cardiovascular medications, including beta-blockers, angiotensin-converting enzyme inhibitors (ACEIs), angiotensin II receptor blockers (ARBs), calcium channel blockers, diuretics, statins, insulin, and oral antidiabetics. The generic names of the pharmacological therapies were recorded.

### 2.6 Outcome measures

Outcomes were measured as the proportion of patients showing adequate CRF control based on the following criteria: BMI < 25, non-somoking, Controlleed LDL-c levels [11,12], controlled blood glucose (<115mg/dL) [13], SBP and DBP <130/80mm Hg and <140/90 mm Hg [14,15], and medication use for the treatment of elevated BP, lipids, and glucose.

### 2.7 Statistical analysis

Descriptive statistics were used to estimate the prevalence rates of CRFs and medication use at the interview and clinical examinations. The patients’ demographics, risk factor profiles, and treatments were described according to unweighted means, standard deviations, and proportions. Prevalence stratified by sex and age (< 60 vs. ≥ 60 years) was compared using Fisher’s exact test. Variables with a statistical significance level of P < 0.10 in univariate logistic regression or with clinical importance, including age ≥ 60 years, sex, hypertension, diabetes, hypercholesterolemia, obesity, smoking, total household income, and education levels were included in the forward logistic multivariate regression analysis. Crude and adjusted odds ratios (OR) and their respective 95% confidence intervals (CI) were calculated and reported. A P-value < 0.05 was considered to indicate statistical significance, and all analyses were two-tailed. All statistical analyses were performed using IBM SPSS Statistics 24.0 software (SPSS 24.0, Chicago, USA).

### 2.8 Ethical considerations

The study was approved by the Brazilian Health Regulatory Agency, The National Research Ethics Commission (CONEP), and all local ethics committees in the participating cities. Written informed consent was obtained from each patient or their legal representative before clinical examinations and interviews.

## 3. Results

### 3.1 Population characteristics

A total of 7,724 subjects with at least one CVD risk factor were interviewed, and clinical visits were evaluated in the BHU. The subjects’ mean (min-max) age was 59.2 (range 18 to 94) years, and 53.7% were aged 60 years or older. A total of 66.7% were women; of the subjects, 96.2% had hypertension, 78.8% had diabetes mellitus type II, and 71.1% had dyslipidemia previously documented in medical records. The prevalence of physical inactivity was 54.3% and that of moderate physical activity (30 minutes or more, three times a week on average) was 13.0% (Table 1).

**Table 1:** Characteristics of the study population.


VARIABLES	ALL SUBJECTS(7724)	MEN (2553)	WOMEN (5153)	P-VALUE

*Age, mean (min-max)*	59.2 (18 – 94)	61.1 (18 – 94)	58.3 (19 – 92)	<0.001

<50 years, % (n)	23.3 (1801)	18.3 (469)	25.7 (1321)	0.217

50–59 years, % (n)	23.0 (1769)	21.0 (535)	24.0 (1235)	0.609

60–69 years, % (n)	32.9 (2541)	34.7 (886)	32.0 (1650)	0.250

≥70 years, % (n)	20.8 (1613)	26.0 (663)	18.3 (947)	0.945

Smoke				

Current smokers, % (n)	20.7 (1596)	21.9 (558)	20.1 (1038)	<0.001

Former smokers, % (n)	38.7 (2987)	48.5 (1238)	33.9 (1749)	<0.001

Hypertension, % (n)	96.2 (7430)	95.1 (2428)	97.1 (5002)	0.085

Dyslipidaemia, % (n)	70.1 (5418)	68.6 (1751)	71.2 (3667)	<0.001

Diabetes, % (n)	78.8 (6083)	78.9 (2014)	79.0 (4069)	0.644

Central obesity, % (n)	59.8 (4617)	37.3 (952)	71.1 (3665)	<0.001

Overweight, % (n)	34.9 (2692)	40.7 (1038)	32.1 (1654)	0.001

Obesity, % (n)	41.7 (3218)	33.0 (842)	46.1 (2376)	<0.001

Physical inactivity defined as no physical activity, irregular or daily physical activity <30 min on average per week, % (n)	54.3 (4188)	50.6 (1287)	56.2 (2901)	<0.001

Moderate physical activity ≥30 min onaverage three times a week, % (n)	13.0 (1001)	14.1 (358)	12.4 (643)	0.562

Daily vegetables and fruits consumption, % (n)	48.3 (3737)	41.6 (1064)	51.8 (2673)	<0.001

*Educational levels, % (n)*				0.192

Illiterate	14.2 (1068)	13.4 (329)	14.9 (739)	

Incomplete elementary school	48.7 (3593)	49.5 (1213)	48 (2380)	

Complete elementary school	12.8 (936)	13.3 (329)	12.3 (607)	

Incomplete high school	4.8 (353)	4.8 (117)	4.8 (236)	

Finished high school	15.9 (1188)	15.8 (390)	16 (798)	

Finished college education	3.6 (277)	3.2 (79)	4 (198)	

*Total household income (USD = R$3.79), quartiles range*				<0.001

≤251.7*	41.5 (3122)	37.3 (896)	45.8 (2212)	

251.8 to 503.4	38.3 (2761)	39 (946)	37.5 (1809)	

503.5 to 1,258.5	17.7 (1212)	20.6 (447)	14.7 (706)	

1,258.6 to 2,517.0	2.1 (143)	2.6 (62)	1.7 (81)	

>2,517.0	0.4 (24)	0.3 (12)	0.3 (12)	


Central obesity: waist circumference ≥88 cm for women and ≥ 102 cm for men; Overweight was defined as body mass index >25 to <30 kg/m^2^; Obesity was body mass index ≥ 30 kg/m^2^. Age presented as mean (min-max); categorical variables presented as percentages and frequencies; * Correspondent to Brazilian Federal Minimum Wage for 2018 fiscal year.

### 3.2 Medication use

The antihypertensive medications taken most frequently by subjects with hypertension were diuretics, followed by ARBs. Most patients were taking one (37.2%) or two (32.5%) antihypertensive medications. Among subjects with diabetes, 34.5% were taking metformin, 8.9% were taking insulin, and 17.7% were using other antidiabetic medications. Among subjects with dyslipidemia, 31.1% were taking statins. The medication intake was similar between the sexes (Table 2).

**Table 2:** Medications use for treatment of patients with hypertension, diabetes mellitus and dyslipidemia.


	ALL SUBJECTS	MEN	WOMEN	P-VALUE*

*Patients using BP-lowering medication, % (n)*				

ACEi	26.9 (1998)	30.3 (735)	25.2 (1263)	0.607

ARBs	37.7 (2803)	37.3 (906)	37.9 (1897)	0.481

Diuretics	44.3 (3290)	39.6 (962)	46.5 (2328)	0.206

Beta-blockers	19.7 (1461)	17.2 (417)	20.9 (1044)	0.173

Calcium-channel blockers	9.5 (706)	9.4 (228)	9.6 (478)	0.666

Other drugs	2.1 (157)	1.9 (46)	2.3 (113)	0.939

*Number of BP lowering drugs, % (n)*				

1 BP lowering drugs	37.2 (2756)	38.5 (936)	36.0 (1820)	0.086

2 BP lowering drugs	32.5 (2418)	30.7 (747)	33.4 (1671)	0.288

3 BP lowering drugs	10.3 (769)	9.1 (222)	11.0 (547)	0.136

≥4 BP lowering drugs	1.7(128)	1.9 (48)	1.6 (80)	0.132

*Patients using glucose-lowering medication, % (n)*				

Metformin	34.5 (2106)	34.0 (684)	34.9 (1422)	0.274

Insulin	8.9 (544)	8.7 (175)	9.0 (369)	0.459

Other oral antidiabetic drugs	17.7 (1076)	18.4 (371)	17.3 (705)	0.355

*Patients using lipid-lowering medications, % (n)*				

Statins	31.1 (1686)	29.9 (489)	32.6 (1197)	0.296


ACEi: Angiotensin converting enzyme inhibitors; ARBs: Angiotensin receptor blockers; BP: Blood pressure. % related to sexes groups. * P-value is related to sexes groups comparations.

### 3.3 Study outcomes

Beyond the three CVD risk factors (hypertension, type 2 diabetes, or dyslipidemia), overweight, obesity, and current smoking were common in our sample. Women showed a greater prevalence of obesity and central obesity compared to men (Table 1).

**Figure 1:**
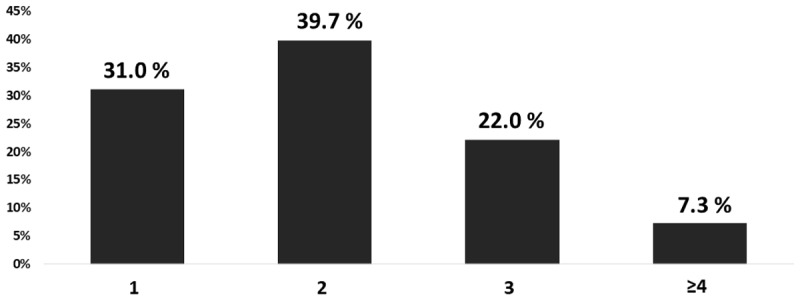
The prevalence of one or more outcomes for the study population.

Figure 1 displays the prevalence of one or more outcomes for the subject. Two and three outcomes accounted for 61.7% of the subjects. Patients with only one CVD risk factor accounted for 31.0% and those with four or more risk factors for 7.3%.

Controlled hypertension was seen in 34.9% with blood pressure readings < 130/80 mmHg and 55.5% with readings < 140/90 mmHg. Among subjects with hypertension using BP-lowering drugs, 33.5% were on target (< 130/80 mmHg). In older subjects (≥ 65 years), BP rates were less controlled (24.3%) compared to younger patients (35.8%). Overall, even among those undergoing recommended BP treatment, almost half of the subjects did not meet their BP target, independent of sex or age. Regarding glucose control, 64.0% of the subjects had a fasting blood glucose < 115 mg/dL. The rate of controlled blood glucose in subjects with known diabetes who were taking antidiabetic medications was 29.5%. The prevalence of subjects achieving target LDL-c levels was 31.8%. Among those with documented dyslipidemia who received any lipid-lowering medication, only 13.9% and 5.2% had an LDL-c < 100 mg/dL and < 70 mg/dl, respectively (Table 3).

**Table 3:** Control CVD risk factors according to patient characteristics.


	ALL SUBJECTS	SEX			AGE		
		
	%, n	MEN(2553)	WOMEN(5153)	P-VALUE	<60 YEARS(3570)	≥60 YEARS(4154)	P-VALUE
	
		%, n	%, n		%, n	%, n	

Systolic/diastolic blood pressure <130/80 mmHg	34.9 (2596)	31.9 (776)	36.4 (1820)	<0.001	35.8 (1283)	24.3 (1257)	0.133

Systolic/diastolic blood pressure <140/90 mmHg	55.5 (4125)	53.8 (1301)	56.4 (2824)	<0.001	54.5 (1946)	50.0 (2080)	0.094

Patients with hypertension using BP lowering and BP <130/80 mmHg	33.5 (2493)	30.3 (737)	35.1 (1756)	0.001	32.8 (1173)	24.3 (1257)	0.082

LDL-c <100 mg/dL	31.8 (844)	35.6 (309)	29.0 (535)	<0.001	30.6 (374)	30.3 (456)	0.719

LDL-c <70 mg/dL	10.1 (276)	12.7 (110)	8.9 (166)	0.003	10.0 (122)	9.9 (149)	0.846

Patients with dyslipidemia using lipids lowering drugs and LDL-c <100 mg/dL	13.9 (132)	15.3 (42)	13.3 (90)	0.019	11.5(44)	15.5 (88)	0.008

Patients with dyslipidemia using lipids lowering drugs and LDL-c <70 mg/dL	5.2 (50)	8.4 (23)	4.0 (27)	<0.001	4.1 (16)	5.9 (34)	0.282

Fasting glucose < 115 mg/dL	64.0 (2136)	61.8 (670)	65.4 (1466)	0.044	67.6 (1028)	61.2 (1071)	<0.001


**Figure 2:**
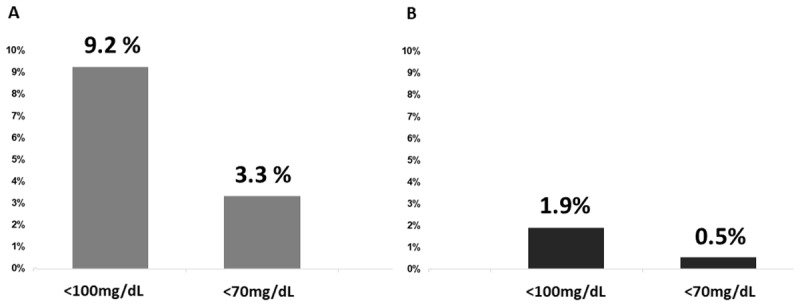
The control of LDL-c is presented for two different targets (LDL-c < 100 mg/dL; < 70 mg/dL) for patients presenting three CVD risk factors (hypertension, dyslipidemia, and diabetes). For all patients, the blood glucose adopted was <115 mg/dL. **A.** Grey: Systolic Blood pressure <140 mm Hg and diastolic blood pressure <90 mmHg. **B.** Dark grey: Systolic Blood pressure <130 mm Hg and diastolic blood pressure <80 mmHg.

The prevalence of controlled CVD risk factors differed between the sexes. Hypertension and diabetes mellitus were more controlled in women than in men; however, men had a higher prevalence of dyslipidemia control. Elderly people had a lower prevalence of diabetes mellitus compared to younger people. However, LDL-c levels < 100 mg/dL were more prevalent among the elderly (Table 3). The control of LDL-c is presented in Figure 2 for two different targets (LDL-c < 100 mg/dL and < 70 mg/dL) for patients presenting three CVD risk factors (hypertension, dyslipidemia, and diabetes). Two different BP targets (< 130/80 mmHg and < 140/90 mmHg) and blood glucose < 115 mg/dL were used. Less than 9.2% of the subjects had an LDL-c < 100 mg/dL when BP < 140/90 mmHg; the proportion was 1.9% once their BP was on target at < 130/80 mmHg. Thus, poor efficiency was found in meeting LDL-c goals when patients showed control of hypertension and diabetes.

### 3.4 Univariate and multivariate analyses

Table 4 describes the clinical and socioeconomic variables associated with the control of CVD risk factors (hypertension, diabetes mellitus, and dyslipidemia). Multivariate analyses revealed that female sex, a diagnosis of hypertension, and obesity were associated with hypertension control. However, education level was associated with greater BP control. On-target glucose and LDL-c levels were associated with diabetes and hypercholesterolemia diagnoses, respectively.

**Table 4:** Association of clinical and social-economics parameters with cardiovascular risk factors on target in univariate and multivariate analyses.


	ODDS RATIO (95% CI)	

	UNIVARIATE ANALYSIS	MULTIVARIATE ANALYSIS

*Blood Pressure < 130 /80 mmHg*		

Sex	0.86 (0.78 – 0.96)	0.64 (0.47–0.88)

Age ≥ 60 y	0.76 (0.69 – 0.84)	

Hypertension	0.31 (0.26–0.37)	0.29 (0.20–0.41)

Diabetes	0.93 (0.83–1.05)	

Hypercholesterolemia	1.10 (0.98–1.24)	

Obesity	0.70 (0.36–0.77)	0.48 (0.36–0.66)

Smoke	1.05 (0.83–1.33)	

Total household income	1.07 (1.04–1.10)	

Educational levels	1.02 (0.96–1.09)	1.10 (1.00–1.21)

*Blood Pressure < 140 /90 mmHg*		

Sex	0.81 (0.73 –0.90)	0.67 (0.49–0.91)

Age ≥ 60 y	0.79 (0.71–0.87)	

Hypertension	0.30 (0.24–0.37)	0.17 (0.10–0.29)

Diabetes	0.88 (0.79–0.99)	0.67 (0.49–0.93)

Hypercholesterolemia	1.20 (1.06–1.35)	

Obesity	0.71 (0.64–079)	0.62 (0.46–0.83)

Smoke	0.84 (0.66–1.06)	

Total household income	1.03 (0.97–1.09)	

Educational levels	1.09 (1.09–1.12)	

*Glucose < 115 mg/dL*		

Sex	0.85 (0.73–0.99)	

Age ≥ 60 y	0.70 (0.60–0.81)	

Hypertension	1.14 (0.90–1.44)	0.51 (0.29–0.91)

Diabetes	0.04 (0.04–0.06)	0.04 (0.02–0.07)

Hypercholesterolemia	0.77 (0.65–0.92)	

Obesity	0.84 (0.73–0.97)	

Smoke	0.57 (0.42–0.77)	0.61 (0.38–0.97)

Total household income	1.04 (0.96–1.13)	

Educational levels	1.04 (1.00–1.10)	

*LDL-C < 100 mg/dL*		

Sex	1.13 (1.14–1.61)	

Age ≥ 60 y	1.04 (0.88–1.22)	

Hypertension	1.16 (0.89–1.52)	

Diabetes	1.67 (1.39–2.00)	2.09 (1.38–3.16)

Hypercholesterolemia	0.64 (0.53–0.57)	0.48 (0.32–0.73)

Obesity	1.16 (0.98–1.37)	

Smoke	1.39 (0.90–1.94)	

Total household income	0.97 (0.89–1.07)	

Educational levels	1.00 (0.96–1.05)	


**Abbreviations:** 95% CI, Confidence interval; *Model with variable selection, considering all significant variables (P < 0.10) in the univariate analysis with at least 90 of information completed. Forward logistic regressions were used to find the significant predictors. Forward logistic regressions were used to find the significant predictors. Sex the male was reference. Obesity was body mass index ≥ 30 kg/m^2^. Familiar monthly income was used ≤ U$ 251.7 as reference. Educational level was used Illiterate as reference.

## 4. Discussion

The EPICO study shows that CRFs are poorly controlled in a large proportion of patients living in Brazilian communities, based on the rates of uncontrolled BP (independent of the BP control criteria adopted), fasting glycemia, and blood cholesterol. The control of risk factors for CVD also differed by sex and age. Even when BP and glucose levels were controlled, patients had poor control of LDL-c levels. In addition, 76.6% of patients were obese or overweight, and only one-quarter had a normal BMI. High rates of central obesity are especially common in women. Brazilian communities have policies for health care access based on the Family Health Program to increase the screening and treatment of non-communicable and infectious diseases. In the Brazilian public health system (Sistema Único de Saúde – SUS), medications for the control of diabetes, hypertension, and dyslipidemia are freely dispensed to all patients, independent of social status, as a strategy to improve population health. However, the EPICO study clearly shows that policies for primary CVD prevention are insufficient for CRF control.

### 4.1 Lifestyle

In our study, most patients showed high rates of components of metabolic syndrome, related to obesity, hypertension, diabetes, and dyslipidemia. Healthy diet habits and weight reduction in overweight and obese patients are recommended to control BP, lipid levels, and diabetes mellitus [12,13,14]. In this study, half of the patients were physically inactive. Moderate physical activity was found in less than 15% of the total study population. These poor outcomes regarding physical activity management among such at-risk patients could be regarded as insufficient lifestyle orientation by health professionals and public policies. In this context, the prevalence of smoking, found in around 20% of the population, was similar to that reported by other registry studies in Europe [5]. Brazil has public policies for tobacco cessation based on elevated taxes and advertising about the risk of tobacco products. A recent study showed that the improvement of smoking cessation programs in communities may be the most cost-effective strategy for preventing CVD [16].

### 4.2 Medication usage and CRF control

BP control rates were low, with less than half of the patients on BP-lowering medication achieving the guideline values. More than 60% of the patients had more than two risk factors, and those receiving specific medication for lowering BP, lipids, and blood glucose were still not achieving their targets. A total of 69.7% of the individuals were taking one or two medications to lower BP, resulting in a BP control of only 33.5% in hypertensive patients. Patients with BP < 130/80 mmHg have a lower incidence of stroke and all-cause death [17]. In the Systolic Blood Pressure Intervention Trial (SPRINT) [18], patients with SBP < 140 mmHg under antihypertensive medications were compared with those with SBP < 120 mmHg using more intensive BP medication, with the latter presenting better results. An SBP lower than 130 mmHg has been related to major cardiovascular events and cardiovascular mortality, especially in subjects with diabetes [19]. In the EPICO study, men and older patients showed poor BP control. A recent Brazilian hypertension registry showed similar rates of BP control in specialized hypertensive outpatient units: 53% were on target, with the majority using diuretic thiazide and ARBs as antihypertensive medications [20]. Data from EPICO reveals an important gap in hypertension management in Brazil, independent of the clinical care setting. Therefore, the findings of the EPICO study support the notion that therapeutic inertia and polypharmacy to control CVD risk factors are far from ideal in primary prevention, regardless of income [21].

Regarding diabetes, our study showed that 64% of the patients were on glycemic target; however, among those using antidiabetic medications, only 29.5% achieved the target. The most frequent antidiabetic drug used was metformin, freely distributed to these patients in BHUs. Recently, Sodium-Glucose Transport Protein 2 (SGLT2) Inhibitors showed good results regarding the reduction of cardiovascular and renal events in diabetic patients and are now available in the public health system for selected patients. Although this will create an opportunity for the better management of high-risk patients [22], in the communities therapeutic inertia is another important issue. New, efficient, and well-tolerated medicines are necessary to increase blood glucose control, but it is also necessary to implement protocols to improve clinical practices. Recently, the benefit of implementing a structured protocol for managing high-risk patients for the reduction of CVD events was demonstrated [23]. These protocols should be urgently implemented in public settings in Brazilian communities. Previous reports of diabetes in Brazil have shown that the rates of diabetes mellitus control are between 22% and 27% in patients with diabetes [24, 25]. The Brazilian Longitudinal Study on Adult Health, the ELSA-Brazil study, showed that 65% of participants with diabetes had two or more comorbidities, indicating a high-risk population [26]. These data are similar to the EPICO study findings, reinforcing the poor control of diabetes in Brazil.

The management of dyslipidemia and achievement of LDL-c targets also deserve attention, with less than 31.1% of all patients taking statins. Of those with documented dyslipidemia, only one-third used statins, with the rates still lower among women. Furthermore, of the patients with dyslipidemia taking statins, only 14% had LDL-c values in the recommended target ranges. The rate of LDL-c target achievement in younger patients was only 10%. In Brazil, the HiperDia program is focused on the management of hypertension and diabetes in BHUs, which are the pivotal centers for community assistance. In this case, the EPICO study showed that the majority of such patients were at high risk, with only a minority exhibiting CRF control. The EPICO study focused on the use of statins because they are the most commonly available lipid-lowering drug type in the community’s basic health system.

The findings of the EPICO study regarding treated patients with dyslipidemia show that the number of subjects on LDL-c target is lower than those in European registries such as EURIKA or EUROASPIRE, which reported values of 41% and 47% for LDL-c < 116 mg/dL and LDL-c < 100 mg/dL, respectively [5, 27]. Other registry studies investigated the achievement of LDL-c targets in patients at risk who received lipid-lowering therapy. The proportion of patients achieving the guideline-recommended treatment target was 44% for LDL-c [28]. In Brazil, even those patients with lower education levels have little knowledge about their recommended LDL-c goals according to their risk or are not receiving the appropriate lipid-lowering therapy [29]. In addition, many patients stop their medication for personal reasons or with the agreement of their physicians despite recent data on primary prevention reinforcing the effectiveness of LDL-c levels below 100 or 70 mg/dL in reducing CVD events [30].

Taken together, adequate treatment for hypertension, diabetes, and dyslipidemia seems crucial for reducing CVD events. Pharmacological and non-pharmacological therapies should be implemented in basic health programs. The World Health Organization has established the HEART program [31] to implement health systems using algorithms for medication prescriptions can will apply to any country within a basic health system. Our results reveal that in the Brazilian scenario, patients have free access to medications; our results also reflect a potential therapeutic inertia that may be overcome based on health policy implementation, including large training programs involving physicians, nurses, pharmacists, community agents, health professionals, and especially, patients. Protocols for pharmacological and non-pharmacological therapies should be implemented within basic health programs in communities through structured-care Familial Health Programs that are well established in BHUs to overcome therapeutic inertia.

### 4.3 Limitations

Limitations may include the criteria for patient inclusion; only those with previous diagnoses of one or more of hypertension, diabetes mellitus type II, and dyslipidemia were included. The study did not use measurements of glycated hemoglobin for the diagnosis and control of diabetes because this laboratory analysis is not routine in primary prevention within the communities of Brazil. The geographical region of São Paulo State, along with the BHUs evaluated in each city, was chosen to increase the representativeness of the study. A major strength of the study is that data were collected using clinical visits with standardized methods, including centralized data management and analyses, rather than from general practice medical records, where the recording of risk factors is usually incomplete.

## 5. Conclusion

In conclusion, the EPICO study provides a unique overview of CRF control in patients at Brazilian community clinics. This study clearly demonstrates that most patients with documented CRF, the control of BP, blood glucose and lipids control is poor with majority of patients not achieving guidelines-recommendations.

## References

[1] Walli-Attaei M, Joseph P, Rosengren A, Chow CK, Rangarajan S, Lear SA, et al., Variations between women and men in risk factors, treatments, cardiovascular disease incidence, and death in 27 high-income, middle-income, and low-income countries (PURE): a prospective cohort study. Lancet. 2020; 396(10244): 97–109. DOI: 10.1016/S0140-6736(20)30543-2 32445693

[2] Gooding HC, Gidding SS, Moran AE, Redmond N, Allen NB, Bacha F, et al. Challenges and opportunities for the prevention and treatment of cardiovascular disease among young adults: Report from a National Heart, Lung, and Blood Institute working group. J Am Heart Assoc. 2020; 9(19): e016115. DOI: 10.1161/JAHA.120.016115 32993438 PMC7792379

[3] Chomistek AK, Chiuve SE, Eliassen AH, Mukamal KJ, Willett WC, Rimm EB. Healthy lifestyle in the primordial prevention of cardiovascular disease among young women. J Am Coll Cardiol. 2015; 65: 43–51. DOI: 10.1016/j.jacc.2014.10.024 25572509 PMC4291551

[4] Barbaresko J, Rienks J, Nothlings U. Lifestyle indices and cardiovascular disease risk: A meta-analysis. Am J Prev Med. 2018; 55: 555–564. DOI: 10.1016/j.amepre.2018.04.046 30241617

[5] Kotseva K, De Backer G, De Bacquer D, Rydén L, Hoes A, Grobbee D, et al. Primary prevention efforts are poorly developed in people at high cardiovascular risk: A report from the European Society of Cardiology EURObservational Research Programme EUROASPIRE V survey in 16 European countries. Eur J Prev Cardiol. 2021; 28(4): 370–379. DOI: 10.1177/2047487320908698 33966079

[6] Fernandes LC, Bertoldi AD, Barros AJ. Health service use in a population covered by the Estratégia de Saúde da Família (Family Health Strategy). Rev Saude Publica. 2009; 43(4): 595–603. DOI: 10.1590/S0034-89102009005000040 19547801

[7] Précoma DB, Oliveira GMM, Simão AF, Dutra OP, Coelho OR, Izar MCO, et al. Updated cardiovascular prevention guideline of the Brazilian Society of Cardiology – 2019. Arq Bras Cardiol. 2019; 113(4): 787–891. DOI: 10.5935/abc.20190204 31691761 PMC7020870

[8] 2019 ACC/AHA guideline on the primary prevention of cardiovascular disease: a report of the American College of Cardiology/American Heart Association Task Force on Clinical Practice Guidelines. Circulation. 2019; 140: e596–e646. DOI: 10.1161/CIR.0000000000000678 30879355 PMC7734661

[9] Williams B, Mancia G, Spiering W, Agabiti Rosei E, Azizi M, Burnier M, et al. ESC/ESH guidelines for the management of arterial hypertension. Eur Heart J. 2018; 39(33): 3021–3104. DOI: 10.1093/eurheartj/ehy339 30165516

[10] Friedewald WT, Levy RI, Fredrickson DS. Estimation of the concentration of low-density lipoprotein cholesterol in plasma, without use of the preparative ultracentrifuge. Clin Chem. 1972; 18: 499–502. DOI: 10.1093/clinchem/18.6.499 4337382

[11] Faludi AA, Izar MCO, Saraiva JFK, Chacra APM, Bianco HT, Afiune A Neto, et al. Atualização da Diretriz Brasileira de Dislipidemias e Prevenção da Aterosclerose – 2017. Arq Bras Cardiol. 2017; 109(2Supl.1): 1–76. DOI: 10.5935/abc.20170121 28813069

[12] Task Force Members; ESC Committee for Practice Guidelines (CPG); ESC National Cardiac Societies. 2019 ESC/EAS guidelines for the management of dyslipidaemias: Lipid modification to reduce cardiovascular risk. Atherosclerosis. 2019; 290: 140–205. DOI: 10.1016/j.atherosclerosis.2019.08.014 31591002

[13] International Diabetes Federation. Clinical Guidelines Task Force global guideline for type 2 diabetes. Brussel; 2012. www.idf.org. (Accessed 20 june 2021).

[14] Authors/Task Force Members:, Piepoli MF, Hoes AW, Agewall S, Albus C, Brotons C, Catapano AL, Cooney MT, et al. 2016 European guidelines on cardiovascular disease prevention in clinical practice. The Sixth Joint Task Force of the European Society of Cardiology and Other Societies on Cardiovascular Disease Prevention in Clinical Practice. Eur Heart J. 2016; 37: 2315–2381. DOI: 10.1093/eurheartj/ehw106 27222591 PMC4986030

[15] Unger T, Borghi C, Charchar F, Khan NA, Poulter NR, Prabhakaran D, et al., 2020 International Society of Hypertension Global Hypertension Practice Guidelines. Hypertension. 2020; 75(6): 1334–1357. DOI: 10.1161/HYPERTENSIONAHA.120.15026 32370572

[16] Akanbi MO, Carroll AJ, Achenbach C, O’Dwyer LC, Jordan N, Hitsman B, et al. The efficacy of smoking cessation interventions in low- and middle-income countries: A systematic review and meta-analysis. Addiction. 2019; 114(4): 620–635. DOI: 10.1111/add.14518 30506845 PMC6411424

[17] Thomopoulos C, Parati G, Zanchetti A. Effects of blood pressure lowering on outcome incidence in hypertension. 1. Overview, meta-analyses, and meta-regression analyses of randomized trials. J Hypertens. 2014; 32(12): 2285–2295. DOI: 10.1097/HJH.0000000000000378 25255397

[18] SPRINT Research Group, Wright, JT Jr, Williamson JD, Whelton PK, Snyder JK, Sink KM, Rocco MV, et al. A randomized trial of intensive versus standard blood-pressure control. N Engl J Med. 2015; 373(22): 2103–2116. DOI: 10.1056/NEJMoa1511939 26551272 PMC4689591

[19] Ettehad D, Emdin CA, Kiran A, Anderson SG, Callender T, Emberson J, et al. Blood pressure lowering for prevention of cardiovascular disease and death: A systematic review and meta-analysis. Lancet. 2016; 387(10022): 957–967. DOI: 10.1016/S0140-6736(15)01225-8 26724178

[20] Lopes, RD, Barroso WKS, Brandao AA, Barbosa ECD, Malachias, MVB, Gomes, MM, et al. The First Brazilian Registry of Hypertension. Am Heart J. 2018; 205: 154–157. DOI: 10.1016/j.ahj.2018.08.012 30268352

[21] Dixon DL, Sharma G, Sandesara PB, Yang E, Braun LT, Mensah GA, et al. Therapeutic inertia in cardiovascular disease prevention: Time to move the bar. J Am Coll Cardiol. 2019; 74(13): 1728–1731. DOI: 10.1016/j.jacc.2019.08.014 31558257

[22] Nelson AJ, Pagidipati NJ, Aroda VR, Cavender MA, Green JB, Lopes RD, et al. Incorporating SGLT2i and GLP-1RA for cardiovascular and kidney disease risk reduction: Call for action to the cardiology community. Circulation. 2021; 144(1): 74–84. DOI: 10.1161/CIRCULATIONAHA.121.053766 34228476

[23] Schwalm JD, McCready T, Lopez-Jaramillo P, Yusoff K, Attaran A, Lamelas P, et al. A community-based comprehensive intervention to reduce cardiovascular risk in hypertension (HOPE 4): A cluster-randomised controlled trial. Lancet. 2019; 394(10205): 1231–1242. DOI: 10.1016/S0140-6736(19)31949-X 31488369

[24] Mendes ABV, Fittipaldi JAS, Neves RCS, Chacra AR, Moreira ED, Jr. Prevalence and correlates of inadequate glycaemic control: Results from a nationwide survey in 6,671 adults with diabetes in Brazil. Acta Diabetol. 2010; 47: 137–145. DOI: 10.1007/s00592-009-0138-z 19655083 PMC2859160

[25] Viana LV, Leitão CB, Kramer CK, Zucatti AT, Jezini, DL, Felício J, et al. Poor glycaemic control in Brazilian patients with type 2 diabetes attending the public healthcare system: A cross-sectional study. BMJ Open. 2013; 3(9): e003336. DOI: 10.1136/bmjopen-2013-003336 PMC3780317 24052610

[26] Coutinho DF, de Figueiredo, RC, Duncan BB, Schmidt MI, Barreto SM, Diniz MFHS. Association between control of diabetes mellitus and polypharmacy at the Brazilian Longitudinal Study of Adult Health (ELSA-Brasil). Pharmacoepidemiol Drug Saf. 2021; 30(6): 749–757. DOI: 10.1002/pds.5236 33772928

[27] Banegas JR, López-García E, Dallongeville J, Guallar E, Halcox JP, Borghi C, et al. Achievement of treatment goals for primary prevention of cardiovascular disease in clinical practice across Europe: The EURIKA study. Eur Heart J. 2011; 32: 2143–2152. DOI: 10.1093/eurheartj/ehr080 21471134 PMC3164103

[28] Blom DJ, Santos RD, Daclin V, Mercier F, Ruiz AJ, Danchin N; ICLPS study group. The challenge of multiple cardiovascular risk factor control outside Western Europe: Findings from the International Cholesterol Management Practice Study. Eur J Prev Cardiol. 2020; 27(13): 1403–1411. DOI: 10.1177/2047487319871735 31533447 PMC7457454

[29] Santos RD, Pereira C, Cesena F, Laurinavicius AG, Tabone, V, Bittencourt MS. Cardiovascular risk misperception and low awareness of familial hypercholesterolemia in individuals with severe hypercholesterolemia. Arq Bras Cardiol. 2021; 116(4): 706–712. DOI: 10.36660/abc.20190516 33566934 PMC8121404

[30] Vallejo-Vaz AJ, Robertson M, Catapano AL, Watts GF, Kastelein JJ, Packard CJ, et al. Low-density lipoprotein cholesterol lowering for the primary prevention of cardiovascular disease among men with primary elevations of low-density lipoprotein cholesterol levels of 190 mg/dL or above: Analyses from the WOSCOPS (West of Scotland Coronary Prevention Study) 5-year randomized trial and 20-year observational follow-up. Circulation. 2017; 136: 1878–1891. DOI: 10.1161/CIRCULATIONAHA.117.027966 28877913

[31] World Health Organization. HEARTS: technical package for cardiovascular disease management in primary health care. Geneva: World Health Organization; 2016. https://www.who.int/cardiovascular_diseases/hearts/Hearts_package.pdf. (Accessed 20 June 2021).

